# Composite Blood-Count Inflammatory Indices in Diabetic Kidney Disease and Advanced, Largely Dialysis-Dependent Non-Diabetic Chronic Kidney Disease: A Single-Centre, Four-Group Cross-Sectional Study

**DOI:** 10.3390/jcm15207910

**Published:** 2026-10-13

**Authors:** Aycan Acet, Neslihan Tezcan

**Affiliations:** 1 Department of Internal Medicine, Faculty of Medicine, Kutahya Health Sciences University, Kutahya 43100, Turkey; 2 Department of Nephrology, Faculty of Medicine, Kutahya Health Sciences University, Kutahya 43100, Turkey; neslihan.tezcan@ksbu.edu.tr

**Keywords:** chronic kidney disease, diabetic kidney disease, dialysis, monocyte-to-lymphocyte ratio, neutrophil-to-lymphocyte ratio, systemic inflammation response index

## Abstract

**Background**/**Objective**: The neutrophil-to-lymphocyte ratio (NLR), monocyte-to-lymphocyte ratio (MLR), and systemic inflammation response index (SIRI) have been proposed as markers of diabetic kidney disease (DKD); however, comparisons are almost always confined to individuals with diabetes mellitus (DM). We examined whether an elevated index is specific to DKD or accompanies chronic kidney disease (CKD) and dialysis of any cause and whether any index adds information to routinely available variables. **Methods**: In this retrospective cross-sectional study, 232 participants were analysed in four groups: 60 controls without DM or kidney disease, 63 with DM alone, 66 with DKD (12 receiving dialysis), and 43 with non-diabetic CKD, a predominantly advanced and dialysis-dependent comparator (32 receiving dialysis; median estimated glomerular filtration rate [eGFR] 7.4 mL/min/1.73 m^2^). Among the 129 participants with DM, each index was added separately to two comparator models defined before the indices were analysed: Model 1, routinely available correlates of DKD; Model 2, variables not determined by renal function (age, sex, glycated haemoglobin [HbA1c], and fasting glucose); And Model 3, fitted for the 109 participants with CKD of either cause, took its cause as the outcome, and was adjusted for age, sex, eGFR, and dialysis status. Contributions were assessed using likelihood ratio block tests with Bonferroni correction for the three indices tested within each model. **Results**: All three indices differed across the groups (*p* < 0.001) and were highest in the non-diabetic CKD group. Within the nephropathy groups, all three indices were higher in participants receiving dialysis, whereas DKD and non-diabetic CKD did not differ significantly within the small strata of comparable severity. No index improved Model 1 (area under the curve [AUC] 0.803; *p* ≥ 0.076) or Model 3 (AUC 0.874; *p* ≥ 0.569). All three improved Model 2 (AUC 0.683; MLR Δχ^2^ = 6.61, *p* = 0.010)**,** but only MLR remained significant after Bonferroni correction, the optimism-corrected gain in AUC was at most 0.033, and no index remained significant after the 12 participants receiving dialysis were excluded (*p* ≥ 0.091). Each index was inversely correlated with eGFR among participants with DM (Spearman’s ρ = −0.31 to −0.37). **Conclusions**: In this single-centre cohort, the indices focused on renal impairment and dialysis, not on diabetes-specific processes. These indices provided no additional insight beyond routine renal impairment markers already included in the model; thus, they were redundant rather than informative. The study design cannot differentiate between DM and non-DM kidney disease in individuals with DM, and these results need validation through prospective, stage-matched studies.

## 1. Introduction

Diabetic kidney disease (DKD) develops in approximately 40% of people with diabetes and is the leading cause of chronic kidney disease (CKD) worldwide, a burden that continues to grow [1,2]. Early recognition of DKD and its distinction from kidney disease of other causes remains a practical challenge in diabetes care. Chronic low-grade inflammation is an established component of its pathogenesis [3]. Hyperglycaemia and advanced glycation end products promote macrophage recruitment, which correlates with serum creatinine and interstitial fibrosis, and oxidative stress and cytokine signalling are closely coupled in this process [4,5]. Interleukin-6 and C-reactive protein are the established circulating markers of this inflammatory state in CKD, but neither is routinely measured in diabetes or nephrology outpatient clinics [6].

Indices derived from complete blood counts add no cost beyond a test already performed and are analytically stable across platforms. In a meta-analysis of 48 studies, 12 that examined the neutrophil-to-lymphocyte ratio (NLR) in nephropathy reported a moderate elevation (standardised mean difference 0.63; 95% confidence interval [CI] 0.43–0.83) [7]. The monocyte-to-lymphocyte ratio (MLR) discriminated nephropathy with an area under the curve (AUC) of 0.874 among 301 patients and independently predicted diabetic kidney injury in the MADKID study [8,9]. A systematic review confirmed the association for NLR (odds ratio 1.84) but not for the platelet-to-lymphocyte ratio or the systemic immune-inflammation index [10]. The systemic inflammation response index (SIRI) has been linked to DKD and a decline in renal function in 9537 patients, although with an AUC of only 0.61 [11,12]. Composite indices have also been associated with nephropathy defined by albuminuria [13]. In population-based analyses of the National Health and Nutrition Examination Survey, SIRI and the systemic immune-inflammation index were associated with prevalent CKD, albuminuria, and reduced estimated glomerular filtration rate (eGFR) among approximately 41,000 adults [14,15], and NLR predicted all-cause mortality among 6880 adults with CKD [16]. The platelet-to-lymphocyte ratio and the systemic immune-inflammation index were not analysed in the present study: a systematic review found no association of either with DKD [10], the largest available cohort judged the systemic immune-inflammation index insufficient for prediction [12], and both incorporate the platelet count, which falls with advancing kidney disease and was therefore reserved for the comparator models described below.

However, three methodological constraints limit this literature. First, comparisons are usually confined to individuals with diabetes, with and without nephropathy. In the absence of a comparator group with non-diabetic CKD, it is not possible to determine whether a high index is due to diabetes or kidney disease from other causes. This distinction is practically significant, as individuals with both diabetes and CKD may have one or both conditions [17]. A four-group design that includes a non-diabetic CKD arm can address the first part of this question, namely whether the elevation of an index is specific to diabetes or accompanies kidney disease of any cause; it cannot determine whether an individual with diabetes and CKD has diabetic or non-diabetic kidney disease, which would require a biopsy-defined or otherwise independently classified cohort. Second, a correlation with nephropathy does not establish that an index adds to routine care or that it adds to the counts from which it was formed. An index is an exact arithmetic function of its components, and collinearity constrains the comparison. Third, the comparator determines what an incremental analysis detects. Anaemia, hyperuricaemia, thrombocytopenia, and weight loss are consequences of advanced kidney disease rather than antecedents of nephropathy; a comparator assembled by univariable screening will therefore contain proxies for the outcome-defining quantity, and a null result will reflect the comparator’s construction as much as the marker’s performance.

Uraemic immune dysfunction is the principal alternative explanation for an elevated index in patients with kidney disease. Uraemia reduces the number and function of lymphoid cells through accelerated apoptosis, whereas myeloid cell numbers are preserved or increased, and monocytes adopt proinflammatory phenotypes [18]. Dialysis adds further stimuli through membrane contact, vascular access, and retention of middle molecules [6]. Because the lymphocyte count is the shared denominator of NLR, MLR, and SIRI, uraemic lymphopenia alone would raise all three indices without any diabetes-specific process, and any study in which the non-diabetic comparator is more advanced or more often dialysis-dependent than the diabetic group must consider this.

This study addressed these constraints in three ways. First, to determine whether the elevation of NLR, MLR, or SIRI is specific to DKD or is instead a general feature of kidney disease regardless of its cause, a CKD group without diabetes was included, and dialysis, which represents an independent inflammatory stimulus that was unevenly distributed across the groups, was examined separately. Second, to establish whether any index adds information beyond routine care and whether this conclusion depends on how the comparator model is constructed, incremental value was tested against two comparator models defined before the indices were analysed: one reproducing the conventional approach and the other restricted to variables not determined by renal function. Third, each index was compared against its own component counts. The indices were compared across four groups (controls without diabetes or kidney disease, diabetic patients without nephropathy, patients with DKD, and patients with CKD unrelated to diabetes), and every measured variable was assigned a defined analytical role before the indices were analysed.

## 2. Materials and Methods

### 2.1. Study Design and Participants

**Figure 1:**
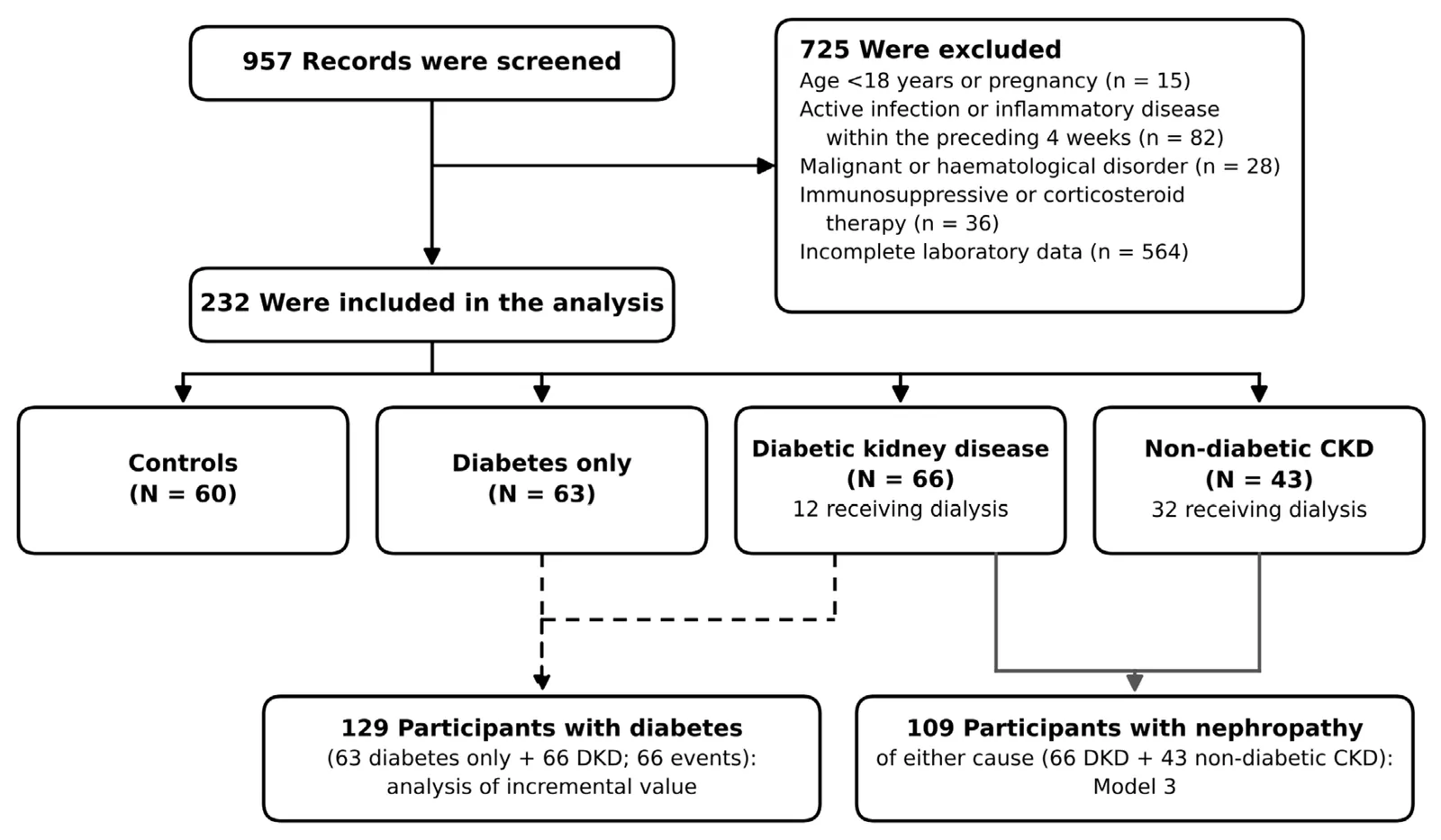
Flow of participants through the study. Of the 957 records screened, 725 were excluded, and 232 participants were analysed in four groups: controls, diabetes only, diabetic kidney disease, and non-diabetic chronic kidney disease (CKD). The 129 participants with diabetes (63 with diabetes only and 66 with diabetic kidney disease; 66 events) formed the population for the analysis of incremental value, and the 109 participants with nephropathy of either cause formed the population for Model 3. Solid black arrows indicate the flow of records from screening to group assignment; dashed arrows indicate the pooling of the diabetes-only and diabetic kidney disease groups into the population for the analysis of incremental value, and grey arrows indicate the pooling of the two nephropathy groups into the population for Model 3.

This retrospective cross-sectional study was conducted at a tertiary care hospital between January 2025 and January 2026. Records were collected from the hospital information system for patients seen in the internal medicine and nephrology outpatient clinics and the haemodialysis unit. The 957 candidate records were identified by ICD-10 diagnosis codes recorded in the hospital information system during the study period: N18 and its subcodes for chronic kidney disease, E11 and its subcodes for type 2 diabetes, and Z00 and its subcodes (general examination without complaint or reported diagnosis) for the control group. All records meeting the eligibility criteria during the study period were reviewed consecutively. The sizes of the four groups represent the number of eligible records in each category, not a predetermined sample size. Outpatient records were reviewed to assemble four groups: controls without diabetes or kidney disease, individuals with type 2 diabetes without nephropathy, individuals with DKD, and individuals with non-diabetic CKD. The control group was selected from patients who presented to the internal medicine outpatient clinics during the same period for any reason or routine health check and who met the eligibility criteria. The protocol required controls to have no diabetes, no known kidney disease, no chronic inflammatory condition, and an eGFR of at least 90 mL/min/1.73 m^2^. In a deviation from this protocol, 20 of the 60 controls had an eGFR between 60 and 89 mL/min/1.73 m^2^ (minimum 61.4). These participants were retained because an eGFR in this range without albuminuria or other evidence of kidney damage does not constitute CKD under the KDIGO definition, so the control group had preserved rather than supranormal renal function; a sensitivity analysis excluding them is reported in Section 3.3. Hypertension, cardiovascular disease, and obesity were not exclusion criteria for any group. The exclusion criteria were age <18 years, pregnancy, active infection or inflammatory disease within the preceding 4 weeks, malignant or haematological disorder, immunosuppressive or corticosteroid therapy, and incomplete laboratory data. The numbers excluded for each reason were age <18 years or pregnancy, *n* = 15; active infection or inflammatory disease, *n* = 82; malignant or haematological disorder, *n* = 28; immunosuppressive or corticosteroid therapy, *n* = 36; incomplete laboratory data, *n* = 564 (Figure 1). Incomplete laboratory data were the largest single reason for exclusion (564 of 957 records, 59%). The variables most often missing were HbA1c and uACR. Because complete laboratory panels are more likely to be available for patients under nephrology or dialysis follow-up than for outpatients attending for a routine check, this exclusion may have introduced selection bias, which is considered in Section Limitations. The study was approved by the institutional ethics committee (2026/09-32 on 27 July 2026) and was conducted in accordance with the Declaration of Helsinki. Data extraction from the medical records began after ethics approval had been obtained, in August 2026; the approval therefore postdates the period during which the clinical data were generated but precedes their use for research. The requirement for informed consent was waived because the analysis used existing medical records.

Diabetes was defined according to the 2025 criteria of the American Diabetes Association and DKD according to the 2022 KDIGO criteria as diabetes with persistent albuminuria (urinary albumin-to-creatinine ratio [uACR] ≥ 30 mg/g) or a sustained eGFR below 60 mL/min/1.73 m^2^ in the absence of another identified kidney disease [19,20]. The KDIGO term ‘diabetic kidney disease’ is used throughout in place of ‘diabetic nephropathy’, because the diagnosis was clinical and no kidney biopsies were performed [20,21]. Persistence was determined from the medical records as at least two abnormal values (uACR ≥ 30 mg/g or eGFR < 60 mL/min/1.73 m^2^) separated by at least three months. In the diabetes-only group, the absence of kidney disease was verified from the same records: all 63 participants had at least two uACR measurements obtained on different occasions, all below 30 mg/g, and nephropathy was excluded on the basis of these values together with the eGFR record. Of the 66 participants with DKD, 52 had a sustained eGFR below 60 mL/min/1.73 m^2^, and persistent albuminuria was confirmed from their earlier records in all 52; the remaining 14 had an eGFR of 60 mL/min/1.73 m^2^ or above with persistent albuminuria that was not explained by another cause. Because the frequency and duration of albuminuria follow-up differed between patients, albuminuria could not be standardised across participants, and only the index values were used for analysis. Non-diabetic CKD was defined using the same functional criteria in the absence of diabetes. The non-diabetic CKD group was identified from the nephrology outpatient clinic and the haemodialysis unit. The recorded clinical diagnosis was hypertensive kidney disease in all 43 participants; no other cause (glomerulonephritis, polycystic kidney disease, obstructive uropathy, or unknown cause) was recorded. By the exclusion criteria, no participant was receiving immunosuppressive or corticosteroid therapy at the time of sampling, and no participant had a recorded diagnosis of an immune-mediated kidney disease that would have called for such treatment in the past.

Of the 957 records screened, 725 were excluded and 232 were analysed (60 controls, 63 with diabetes alone, 66 with DKD, and 43 with non-diabetic CKD) (Figure 1). Dialysis status, a covariate defined before the analysis, was recorded for each participant: 12 of 66 (18.2%) with DKD and 32 of 43 (74.4%) with non-diabetic CKD were receiving maintenance dialysis; no other participant was. All participants receiving dialysis were treated with haemodialysis. Blood samples were obtained before a mid-week haemodialysis session; the vascular access was an arteriovenous fistula in all 44 participants receiving dialysis, and the median dialysis vintage was 18 months (IQR 9.0 to 28.5). Patients dialysed through a tunnelled central venous catheter were not included: exit-site skin inflammation, recent catheter-related infection, and neutrophilic leukocytosis are frequent in this group and fell under the exclusion criterion of active infection or inflammatory disease, and their presence would have confounded the interpretation of the indices. Data on erythropoiesis-stimulating agents and intravenous iron were not retrievable. The two nephropathy groups therefore differed substantially in severity: median eGFR was 35.5 mL/min/1.73 m^2^ in DKD and 7.4 in non-diabetic CKD, and the non-diabetic group functioned as an advanced, largely dialysis-dependent comparator rather than a stage-matched one (KDIGO G categories are reported in Section 3.1).

### 2.2. Laboratory Measurements

Complete blood counts were obtained after an overnight fast and analysed on a Sysmex XN-1000 automated haematology analyser (Sysmex Corporation, Kobe, Japan) within 2 h of venipuncture; all samples were run on the same instrument under the laboratory’s routine internal and external quality control. Biochemical measurements were performed on the c 702 clinical chemistry module of a Cobas 8000 modular analyser series (Roche Diagnostics, Mannheim, Germany); absolute counts are expressed as ×10^9^/L. Three indices were derived: NLR (neutrophils divided by lymphocytes), MLR (monocytes divided by lymphocytes), and SIRI (neutrophils × monocytes divided by lymphocytes). SIRI is reported in ×10^9^/L rather than cells per microlitre for comparability with published thresholds. The eGFR was calculated using the 2021 CKD-EPI creatinine equation [22]. Because creatinine-based eGFR is not valid in people receiving maintenance dialysis, in whom serum creatinine depends on the timing of the last session and on the dialysis dose, eGFR is reported descriptively for participants not receiving dialysis, participants receiving dialysis are classified as KDIGO category G5D, and the eGFR-based analyses are handled as described in Section 2.3.

Because the three indices shared the lymphocyte count in their denominator, they were analysed individually and never entered the same model. Their component counts and total white cell count were excluded from both comparator models, irrespective of univariable significance, and were reserved for the separate component-count comparison described below. Creatinine and eGFR, constituents of the case definition, were excluded as circular and were reported descriptively only.

### 2.3. Statistical Analysis

Continuous variables are presented as medians (interquartile ranges) and were compared across the four groups using the Kruskal–Wallis test (epsilon-squared as the effect size), followed by Dunn’s post hoc test with Bonferroni correction. Categorical variables were compared using the chi-square test. Because the groups differed in age and sex, the four-group comparison of each index was repeated using rank analysis of covariance adjusted for both (partial eta squared as an effect size). Effect sizes for two-group comparisons were reported as the Hodges–Lehmann estimate of the median difference with a distribution-free 95% CI, together with the rank-biserial correlation (rrb) from the Mann–Whitney test.

The incremental analysis was confined to participants with diabetes (66 events among 129 participants) and used two comparator models whose composition was fixed before the indices were analysed. Model 1 followed the conventional approach: the 13 eligible candidate covariates were age, sex, BMI, waist circumference, fasting glucose, HbA1c, uric acid, aspartate aminotransferase, alanine aminotransferase, haemoglobin, haematocrit, platelet count, and mean platelet volume; the index components (white-cell, neutrophil, lymphocyte, and monocyte counts) and the constituents of the case definition (creatinine and eGFR) were ineligible. A variable entered the model if univariably associated with the outcome (*p* < 0.05), clinically plausible as a correlate of DKD, and not strongly correlated with a variable already retained (Spearman |ρ| > 0.8); platelet count and mean platelet volume were eligible, being reported on the same blood count but forming no part of the indices. Because univariable screening yields optimistic models, the selection was repeated as a sensitivity analysis with LASSO-penalised logistic regression on the same 13 candidates (standardised covariates; penalty chosen by 10-fold cross-validation with the one-standard-error rule), and each index was added to the LASSO-selected model refitted without penalty.

Model 2 was defined on causal grounds, independent of the outcome of Model 1 selection, as the set of routinely available variables that are not determined by renal function: age, sex, HbA1c, and fasting glucose. The rationale was that anaemia, hyperuricaemia, thrombocytopenia, and, in advanced disease, loss of body mass are consequences of reduced glomerular filtration and act as partial proxies for the outcome-defining quantity. In the event, four of the five variables retained in Model 1 were correlated with eGFR in this sample: uric acid, haemoglobin, and platelet count, which are consequences of reduced filtration, and age, which is a determinant of eGFR rather than a consequence of nephropathy and was therefore retained in Model 2; BMI was not correlated with eGFR here (Section 3.2) and was treated as renally influenced on causal rather than empirical grounds. HbA1c and fasting glucose are not wholly independent of renal function in advanced CKD, where anaemia, shortened red-cell survival, erythropoiesis-stimulating agents, and reduced insulin clearance alter both; this is acknowledged as a limitation of Model 2 rather than as a reason to add renal variables to it. Sex was included despite falling just short of the univariable threshold, since eligibility was defined by causal role rather than significance. Waist circumference and BMI were examined in sensitivity analyses rather than in the primary model because BMI reflects protein–energy wasting in advanced CKD. The Spearman correlation of every covariate with eGFR documented how far each comparator encodes renal function, and the discrimination of creatinine alone was estimated as an upper bound.

Model 3 posed the same question to the 109 participants who already had nephropathy, with its cause as the outcome. Because the case definition here is the presence of diabetes rather than the level of renal function, eGFR and dialysis status were confounders rather than circular constituents and were retained alongside age and sex; HbA1c and fasting glucose were excluded as definitional. Since non-diabetic CKD is defined by the absence of diabetes, Model 3 assessed whether the indices could differentiate CKD with diabetes from CKD without diabetes; however, it could not identify whether a person with diabetes had diabetic or non-diabetic kidney disease. Because creatinine-based eGFR is not valid in participants receiving dialysis, Model 3 was fitted in two forms: the primary form, with eGFR and dialysis status as covariates, and a form in which eGFR was treated as not applicable in participants receiving dialysis (dialysis entered as an indicator and eGFR entered only for participants not receiving dialysis). Model 3 was repeated after exclusion of participants receiving dialysis, after restriction to an eGFR below 30 mL/min/1.73 m^2^, and with age and sex alone, to show what the indices reflect when renal function is uncontrolled. The repetition after exclusion of participants receiving dialysis contained only 11 participants with non-diabetic CKD (approximately 3 events per variable) and is presented as descriptive only.

Each index was added as a separate second block to each of the three comparator models. Its contribution was quantified by the likelihood-ratio block test (Δχ^2^, 1 df), the change in Nagelkerke’s R^2^, and the change in the AUC, with paired AUCs compared by DeLong’s test; because DeLong’s test is less sensitive, the block test was the inference of record. Since three indices were tested within each model, block-test *p* values were corrected by both the Bonferroni method (α = 0.0167) and the Benjamini–Hochberg false discovery rate. Odds ratios are reported per clinically meaningful unit: per unit for NLR and SIRI and per 0.1 for MLR; age is reported per 10 years and platelet count per 10 × 10^9^/L throughout. Confidence intervals were not adjusted for multiplicity.

The second comparison assessed each index against its own component counts, entered as one block with the index as the second because the resulting collinearity was arithmetic rather than empirical; only the block test was interpreted.

Discrimination and calibration were corrected for optimism using bootstrap resampling with 1000 replications. The events per variable (EPV) were calculated as the number of outcome events divided by the number of model degrees of freedom, with the smaller outcome group taken as the event count in Model 3. The calibration slopes refer to the optimism-corrected slope of the linear predictor from the bootstrap and are reported for each comparator model with and without each index (Section 3.2). The Hosmer–Lemeshow test was computed but not used for inference owing to its sensitivity to the number of groups chosen (*p* = 0.042 to *p* = 0.878 in Model 1).

Sensitivity analyses comprised the exclusion of participants receiving dialysis (with adjusted odds ratios and CIs reported for every index in both models), repetition of the four-group comparison after exclusion of the 20 controls whose eGFR was between 60 and 89 mL/min/1.73 m^2^**,** the addition of waist circumference and separately, BMI to Model 2, removal of age from Model 2 (its only covariate correlated with eGFR), repetition of Model 1 on a reduced covariate set and with sex forced in, re-comparison of the two nephropathy groups within overlapping severity strata (eGFR < 30 and <15 mL/min/1.73 m^2^) and among participants not receiving dialysis, a comparison restricted to participants with an eGFR of at least 60 mL/min/1.73 m^2^ (albuminuria-defined DKD versus diabetes without kidney disease), which is the only contrast free of renal-function confounding and is reported descriptively, and the Spearman correlation of each index and its component counts with eGFR among participants with diabetes. Hodges–Lehmann differences with 95% CIs are reported for every stratified contrast.

No post hoc power calculation was reported because such calculations add nothing to the confidence intervals, which convey the precision of each estimate. Analyses were performed using IBM SPSS Statistics for macOS, version 31.0 (IBM Corp., Armonk, NY, USA) and Python version 3.12 (Python Software Foundation, Wilmington, DE, USA), along with the pandas (version 2.2.3), SciPy (version 1.14.1), statsmodels (version 0.14.4), and scikit-learn (version 1.5.2) libraries. Statistical significance was determined using a two-tailed *p*-value of <0.05.

## 3. Results

### 3.1. Participants and Composite Indices

Of the 957 records screened, 232 were analysed (Figure 1; Table 1). The groups differed in age, highest in DKD (median 62.0 years; interquartile range [IQR] 56.0 to 71.0), and BMI, lowest in non-diabetic CKD (*p* ≤ 0.002 for both), but not in the proportion of women (*p* = 0.13). Fasting glucose and HbA1c were higher in the two groups with diabetes than in controls and non-diabetic CKD (*p* < 0.001) and did not differ between the diabetes groups. KDIGO G categories are shown in Table 1. Among participants not receiving dialysis, median eGFR was 102.2 mL/min/1.73 m^2^ (IQR 86.8 to 107.5) in controls, 101.2 (85.9 to 109.0) in diabetes only, 40.4 (21.9 to 59.7) in the 54 with DKD and 11.5 (10.2 to 16.6) in the 11 with non-diabetic CKD; across all participants, median eGFR was 35.5 (12.5 to 57.3) in DKD and 7.4 (5.2 to 9.5) in non-diabetic CKD. White-cell and neutrophil counts were highest in the two groups with diabetes; haemoglobin, haematocrit, and platelet counts were lowest in the two groups with nephropathy; and lymphocyte counts were lowest in non-diabetic CKD (*p* < 0.001 for all). Mean platelet volume did not differ (*p* = 0.74).

**Figure 2:**
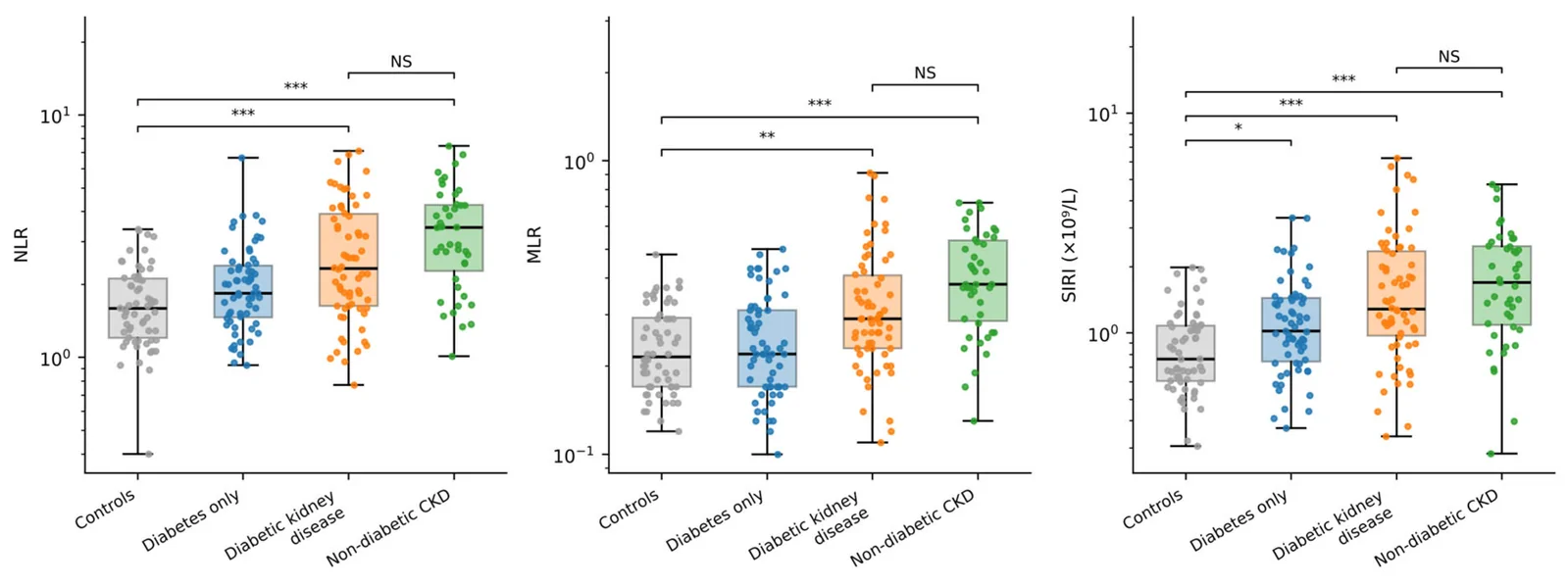
Composite inflammatory indices according to study group. Box plots show the median (horizontal line) and interquartile range (box) of the neutrophil-to-lymphocyte ratio (NLR), monocyte-to-lymphocyte ratio (MLR), and systemic inflammation response index (SIRI) in controls (*N* = 60), diabetes only (*N* = 63), diabetic kidney disease (*N* = 66), and non-diabetic chronic kidney disease (CKD; *N* = 43); whiskers show the extent of the remaining observations, individual values are overlaid, and the vertical axes are logarithmic. Brackets indicate Dunn’s post hoc comparisons with Bonferroni correction: * *p* < 0.05; ** *p* < 0.01; *** *p* < 0.001; NS, not significant.

All three indices differed across the four groups (*p* < 0.001 for all; ε^2^ 0.18 to 0.19) and increased progressively with renal involvement, reaching their highest values in non-diabetic CKD rather than in DKD. The median NLR rose from 1.59 in controls to 3.43 in non-diabetic CKD, MLR from 0.21 to 0.38, and SIRI from 0.76 to 1.70 × 10^9^ cells/L, with intermediate values in the two groups with diabetes (Table 2; Figure 2). In Dunn’s post hoc comparisons, both nephropathy groups differed from the control group for every index (corrected *p* ≤ 0.002) but not from each other (*p* = 0.14 for NLR, 0.06 for MLR, and 1.00 for SIRI); diabetes without nephropathy differed from the control group for SIRI alone (corrected *p* = 0.042). In the unstratified two-group comparison, NLR and MLR were higher in non-diabetic CKD than in DKD (Hodges–Lehmann difference, DKD minus non-diabetic CKD, −0.63, 95% CI −1.25 to −0.06, and −0.09, −0.14 to −0.03, respectively), whereas SIRI was not (−0.21 × 10^9^/L, −0.57 to 0.15)). The four-group differences persisted after adjusting for age and sex (*p* < 0.001; partial η^2^ = 0.17–0.18).

### 3.2. Comparator Models and Incremental Value of the Indices

Six of the 13 eligible covariates were univariably associated with nephropathy at *p* < 0.05, and sex fell just short of the threshold (*p* = 0.056); haematocrit was excluded because it was strongly correlated with haemoglobin (ρ = 0.95) (Table 3). Model 1 comprised age, BMI, uric acid, haemoglobin, and platelet count, and it discriminated well (AUC 0.803; 95% CI 0.728 to 0.872), with every covariate except age contributing independently (Table 4, Panel A). Four of the five (age, uric acid, haemoglobin, and platelet count) were correlated with eGFR among participants with diabetes (|ρ| = 0.23 to 0.42; *p* ≤ 0.007), whereas BMI was not (*p* = 0.068). Creatinine alone discriminated nephropathy with an AUC of 0.931 and eGFR alone with an AUC of 0.935. LASSO selection with the one-standard-error rule retained nine covariates (age, sex, BMI, waist circumference, fasting glucose, uric acid, aspartate aminotransferase, haemoglobin, and platelet count; apparent AUC 0.832 after unpenalised refitting); added to this model, no index was significant (Δχ^2^ 0.76 to 2.23; *p* = 0.136 to 0.383), in agreement with the Model 1 result. Model 2 comprised age, sex, HbA1c, and fasting glucose, none of which was correlated with eGFR apart from age (*p* ≥ 0.15); it discriminated modestly (AUC 0.683; 95% CI 0.583 to 0.768), with only age contributing independently (OR 1.61 per 10 years; 95% CI 1.12 to 2.31; *p* = 0.011) (Table 4, Panel C).

**Figure 3:**
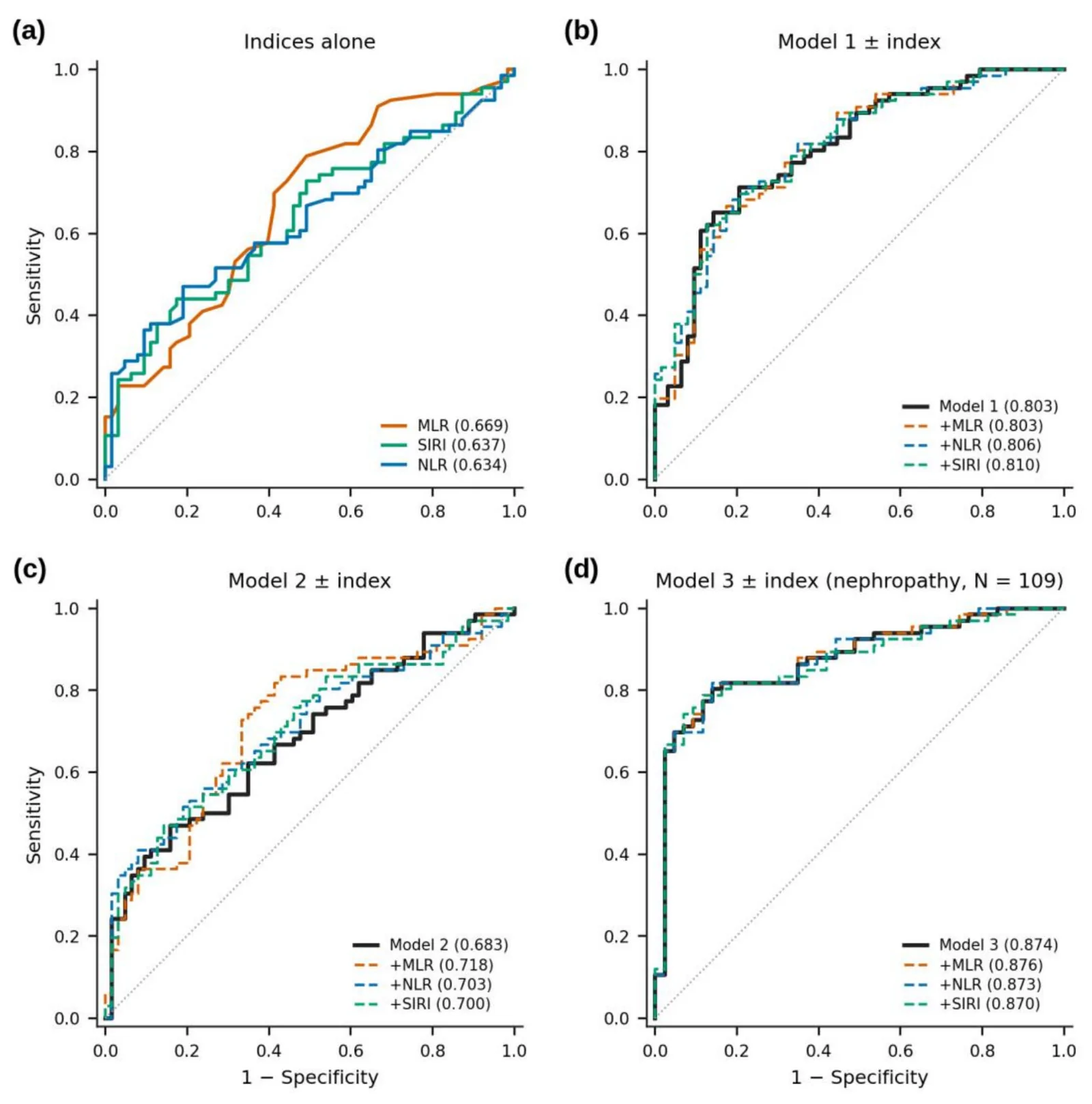
Receiver-operating-characteristic curves for the indices and comparator models. (**a**) Each index alone as a classifier of diabetic kidney disease among the 129 participants with diabetes. (**b**) Model 1 (age, body mass index, uric acid, haemoglobin, and platelet count) with and without each index. (**c**) Model 2 (age, sex, HbA1c, and fasting glucose) with and without each index. (**d**) Model 3 (age, sex, eGFR, and dialysis status), fitted in the 109 participants with nephropathy of either cause, with the cause of nephropathy as the outcome, with and without each index. Values in parentheses are areas under the curve; solid black lines show each comparator model alone, dashed coloured lines show the model with each index added, and the dotted grey diagonal is the line of no discrimination. eGFR, estimated glomerular filtration rate; HbA1c, glycated haemoglobin; MLR, monocyte-to-lymphocyte ratio; NLR, neutrophil-to-lymphocyte ratio; SIRI, systemic inflammation response index.

**Figure 4:**
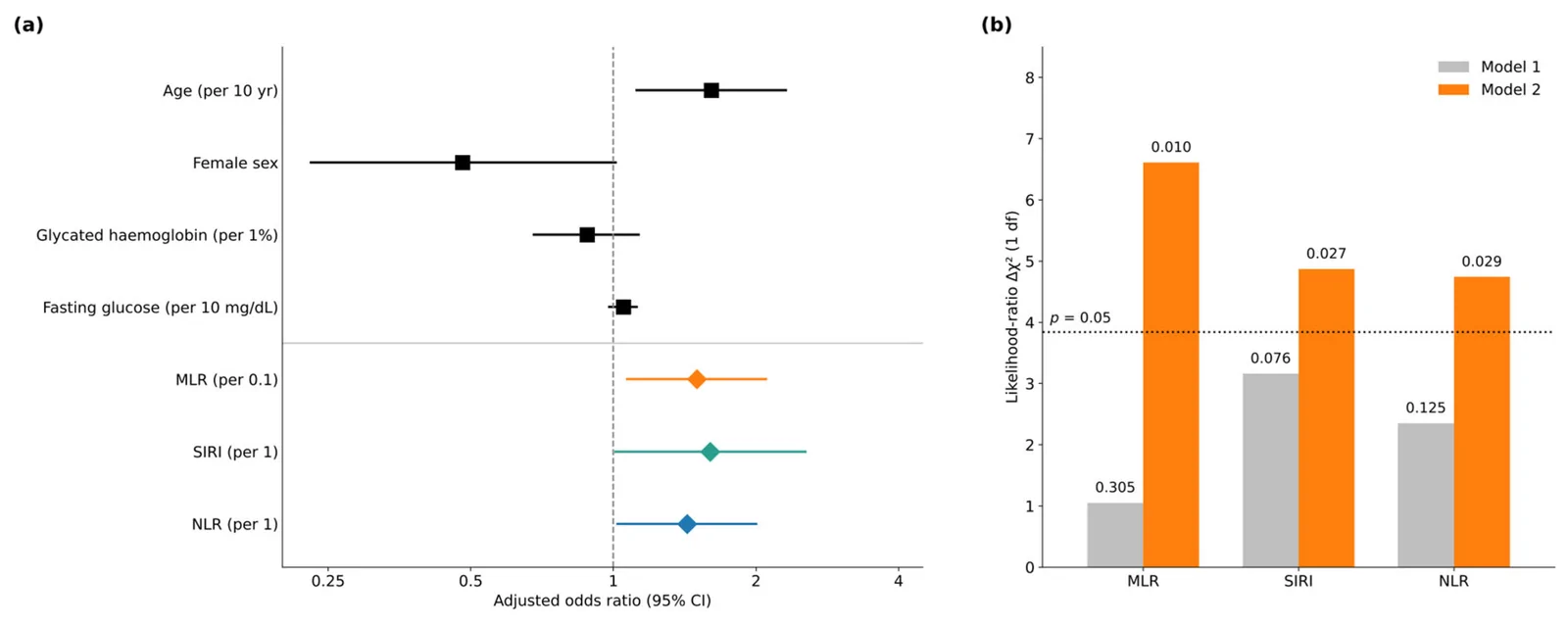
Adjusted associations and incremental values of the indices. (**a**) Adjusted odds ratios with 95% confidence intervals (CI) for the covariates of Model 2 (squares) and for each index added separately to Model 2 (diamonds), plotted on a logarithmic scale; the dashed vertical line marks an odds ratio of 1, and the horizontal grey line separates the covariates of Model 2 from the indices. Odds ratios are per 10 years for age, per 1% for glycated haemoglobin, per 10 mg/dL for fasting glucose, per 0.1 for MLR and per 1 for SIRI and NLR. (**b**) Likelihood ratio Δχ^2^ (1 df) for each index added as a second block to Model 1 (grey) and Model 2 (orange); the nominal *p* value is shown above each bar, and the dotted line marks the value corresponding to *p* = 0.05. MLR, monocyte-to-lymphocyte ratio; NLR, neutrophil-to-lymphocyte ratio; SIRI, systemic inflammation response index.

The two comparator models yielded conflicting results (Table 4; Figure 3 and Figure 4). No index added information to Model 1 (Δχ^2^ 1.05 to 3.16; *p* = 0.076 to 0.305; corrected *p* > 0.18; ΔAUC 0.000 to +0.007; DeLong *p* ≥ 0.56). All three improved Model 2: Δχ^2^ was 6.61 for MLR (*p* = 0.010), 4.87 for SIRI (*p* = 0.027), and 4.74 for NLR (*p* = 0.029), each with a false discovery rate–adjusted *p* of 0.029, although only MLR remained significant after Bonferroni correction (*p* = 0.030). Adjusted odds ratios were 1.50 (95% CI 1.07 to 2.10) per 0.1 increase in MLR, 1.60 (1.01 to 2.54) per unit of SIRI, and 1.43 (1.02 to 2.00) per unit of NLR. Nagelkerke’s R^2^ rose by 0.044 to 0.060 and the AUC from 0.683 to between 0.700 and 0.718, differences that DeLong’s test did not detect (*p* = 0.22 to 0.44) (Table 4, Panel D). Variance inflation factors did not exceed 2.30. After exclusion of the 12 participants receiving dialysis (117 participants; 54 events), no index remained significant in either model: in Model 2, the adjusted odds ratios were 1.23 (95% CI 0.87 to 1.74) per 0.1 for MLR, 1.44 (0.92 to 2.24) per unit for SIRI and 1.32 (0.92 to 1.90) per unit for NLR (Δχ^2^ 1.47 to 2.86; *p* = 0.091 to 0.226), and in Model 1 they were 1.06 (0.73 to 1.55), 1.41 (0.88 to 2.25) and 1.27 (0.88 to 1.83) (*p* = 0.145 to 0.765) (Table 5, Panel B). Among participants with diabetes, each index was inversely correlated with eGFR (Spearman ρ −0.31 for NLR, −0.37 for MLR and −0.34 for SIRI; *p* < 0.001 for all), as was the lymphocyte count positively (ρ 0.30; *p* < 0.001), whereas the neutrophil (ρ −0.06), monocyte (−0.14) and white-cell (0.02) counts were not; the correlations persisted among the 117 participants not receiving dialysis (ρ −0.25 to −0.30) (Table 6, Panel B). As univariable classifiers, the indices discriminated modestly (AUC 0.669 for MLR, 0.637 for SIRI, and 0.634 for NLR) (Figure 3a), and each was higher in participants with nephropathy in an unadjusted comparison (*p* ≤ 0.009 for all) (Table 2 and Table 3).

No index improved its own component counts within Model 1: the counts entered as a block yielded Δχ^2^ values of 0.66 to 2.89 (*p* = 0.24 to 0.72), and each index added to its own components yielded *p* = 0.182 to 0.267. Within Model 2, the counts as a block again showed no improvement (Δχ^2^ 2.68 to 4.05; *p* = 0.13 to 0.26), whereas each index added to its own components approached but did not reach significance on a single degree of freedom (Δχ^2^ = 3.40 for MLR, *p* = 0.065; 3.38 for NLR, *p* = 0.066; 3.27 for SIRI, *p* = 0.071). Variance inflation factors of 5.55 to 6.66 and 7.9 to 9.0 events per variable confined inference in these models to the block test; therefore, the comparison is inconclusive.

Among the 109 participants with pre-existing nephropathy, Model 3 discriminated the underlying cause well (AUC 0.874; 95% CI 0.795 to 0.936; Nagelkerke R^2^ 0.509), attributable primarily to eGFR (odds ratio 2.25 per 10 mL/min/1.73 m^2^; 95% CI 1.20 to 4.20; *p* = 0.011) rather than age, sex, or dialysis status (*p* ≥ 0.21); as noted in the Methods, this reflects the sampling of the two groups, in which eGFR was much lower in non-diabetic CKD, as much as any biological difference. No index added information: Δχ^2^ was 0.32 for SIRI (*p* = 0.569), 0.02 for NLR (*p* = 0.888), and 0.01 for MLR (*p* = 0.913), with adjusted odds ratios of 1.14, 0.98, and 1.02 and changes in AUC of −0.005 to +0.001 (Table 4, Panels E and F; Figure 3d). When eGFR was treated as not applicable in participants receiving dialysis, the model discriminated similarly (AUC 0.862; eGFR among participants not receiving dialysis, odds ratio 2.07 per 10 mL/min/1.73 m^2^, 95% CI 1.16 to 3.71; dialysis, 0.63, 0.13 to 3.00) and again, no index added information (Δχ^2^ 0.00 to 0.22; *p* ≥ 0.64) (Table 4, Panel G). The null persisted after restriction to an eGFR below 30 mL/min/1.73 m^2^ (72 participants; *p* = 0.54 to 0.84). Among the 65 participants not receiving dialysis, of whom only 11 had non-diabetic CKD (approximately 3 events per variable), the corresponding estimates are descriptive only (*p* = 0.17 to 0.95). With only age and sex adjusted, NLR and MLR reached significance (odds ratio 0.76 per unit of NLR, 95% CI 0.58 to 0.99, *p* = 0.036; 0.72 per 0.1 of MLR, 0.56 to 0.93, *p* = 0.009), so that a higher index indicated a non-diabetic rather than a diabetic cause.

Optimism correction lowered the AUCs: the apparent gains over Model 1 disappeared (model 0.803 to 0.776; largest corrected index gain 0.003), whereas those over Model 2 persisted in reduced form (model 0.683 to 0.644; 0.033 for MLR, 0.016 for NLR, 0.014 for SIRI) (Table 5, Panel A). The optimism-corrected calibration slopes were 0.856 for Model 1 (0.832 to 0.837 with each index), 0.780 for Model 2 (0.766 to 0.777 with each index), and 0.781 for Model 3 (0.753 to 0.756 with each index).

### 3.3. Sensitivity Analyses

The loss of Model 2 signal after exclusion of participants receiving dialysis is reported in Section 3.2 (Table 5, Panel B). Adding waist circumference (AUC 0.699) or BMI (AUC 0.693) to Model 2 left all three indices significant (*p* = 0.020 to 0.049), and removing age strengthened them (*p* = 0.002, 0.006, and 0.009 for MLR, SIRI, and NLR, respectively). Repeating Model 1 on a reduced covariate set (age, BMI, haemoglobin, and uric acid; AUC 0.766) or with sex forced in did not alter its null result (*p* ≥ 0.082). Exclusion of the 20 controls with an eGFR of 60 to 89 mL/min/1.73 m^2^ (40 controls remaining; 212 participants) did not alter the four-group comparison: all three indices still differed across the groups (Kruskal–Wallis H 37.7 for NLR, 42.1 for MLR, and 37.8 for SIRI; *p* < 0.001 for all; ε^2^ 0.17 to 0.19), both nephropathy groups still differed from the controls for every index (Dunn corrected *p* ≤ 0.004) but not from each other (*p* = 0.13 for NLR, 0.059 for MLR, and 1.00 for SIRI), and the median values of the 40 remaining controls were unchanged (NLR 1.60, MLR 0.22, SIRI 0.79 × 10^9^/L). The only difference from the full analysis was that the marginal difference between diabetes only and controls for SIRI (corrected *p* = 0.042 in the full sample) was no longer significant (*p* = 0.069).

**Figure 5:**
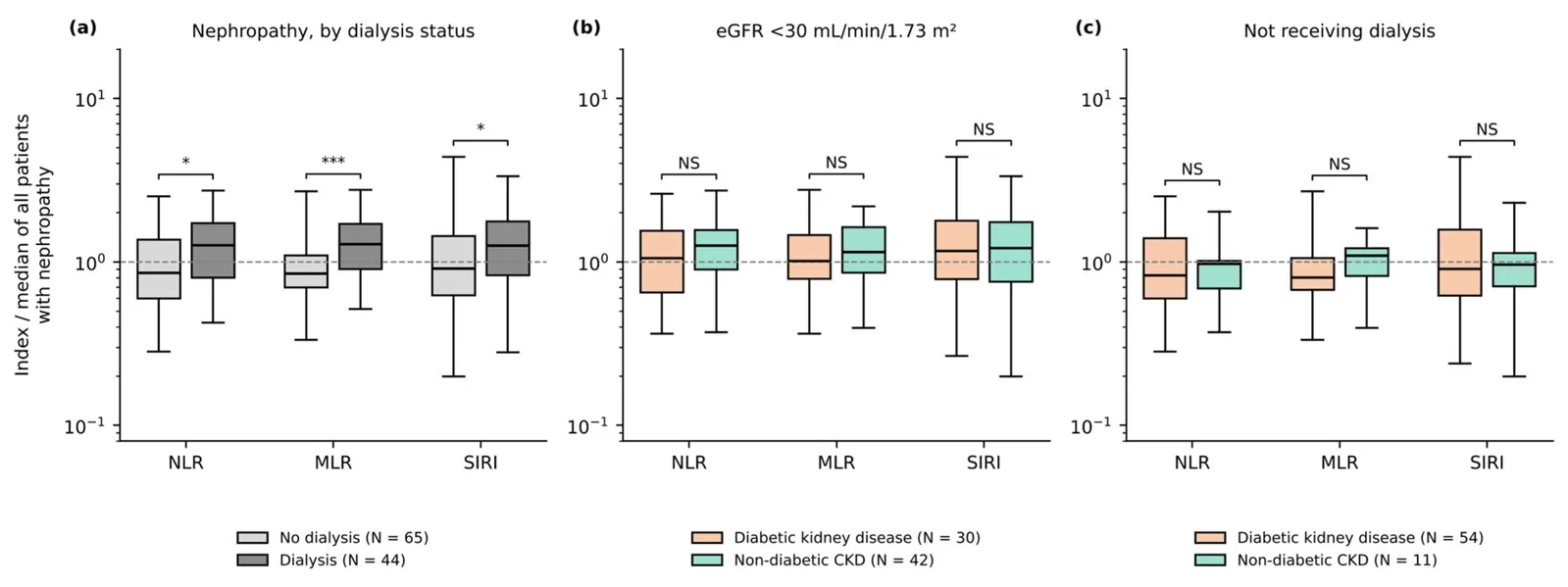
Composite inflammatory indices among the participants with nephropathy according to dialysis status and severity of renal impairment. Each index is expressed relative to the median of all 109 participants with nephropathy (dashed line at 1.0) on a logarithmic scale; boxes show the median and interquartile range, and whiskers show the extent of the remaining observations. (**a**) Participants with nephropathy of either cause receiving dialysis (*N* = 44) versus those not receiving dialysis (*N* = 65). (**b**) Diabetic kidney disease (*N* = 30) versus non-diabetic chronic kidney disease (CKD; *N* = 42) among participants with an eGFR below 30 mL/min/1.73 m^2^. (**c**) Diabetic kidney disease (*N* = 54) versus non-diabetic CKD (*N* = 11) among participants not receiving dialysis. * *p* < 0.05; *** *p* < 0.001; NS, not significant. Hodges–Lehmann differences with 95% confidence intervals for each contrast are given in Table 6. eGFR, estimated glomerular filtration rate; MLR, monocyte-to-lymphocyte ratio; NLR, neutrophil-to-lymphocyte ratio; SIRI, systemic inflammation response index.

Among the 109 participants with nephropathy, all three indices were higher in those receiving dialysis (*N* = 44) than in those who were not (*N* = 65): MLR, 0.42 versus 0.28 (Hodges–Lehmann difference 0.12, 95% CI 0.06 to 0.19; *p* < 0.001; rrb = 0.46); NLR, 3.46 versus 2.34 (0.79, 0.19 to 1.46; *p* = 0.010; rrb = 0.29); and SIRI, 1.79 versus 1.30 × 10^9^/L (0.37, 0.02 to 0.74; *p* = 0.034; rrb = 0.24) (Figure 5a; Table 6, Panel A). The platelet count did not differ by dialysis status (median 209 versus 221 × 10^9^/L; Hodges–Lehmann difference −15, 95% CI −40 to 10; *p* = 0.21), whereas the lymphocyte count (1.31 versus 2.04 × 10^9^/L; −0.60, −0.88 to −0.31; *p* < 0.001), white-cell count (6.50 versus 7.99; *p* = 0.002), and haemoglobin (11.1 versus 13.0 g/dL; *p* < 0.001) were lower with dialysis; the monocyte count did not differ (*p* = 0.76). No index differed significantly between the two nephropathy groups restricted to participants not receiving dialysis (*N* = 54 and 11; *p* = 0.15 to 0.87), despite median eGFRs of 40.4 and 11.5 mL/min/1.73 m^2^, or restricted to an eGFR below 30 (*N* = 30 and 42; *p* = 0.37 to 0.94; |rrb| ≤ 0.13) or below 15 mL/min/1.73 m^2^ (*N* = 20 and 40; *p* ≥ 0.33) (Figure 5b,c). The Hodges–Lehmann differences (DKD minus non-diabetic CKD) were below 30 mL/min/1.73 m^2^: NLR −0.29 (95% CI −1.07 to 0.46), MLR −0.04 (−0.11 to 0.04), SIRI 0.01 (−0.46 to 0.44); below 15: NLR −0.33 (−1.27 to 0.52), MLR −0.01 (−0.11 to 0.07), SIRI 0.02 (−0.54 to 0.45); and among participants not receiving dialysis: NLR −0.10 (−0.85 to 0.84), MLR −0.06 (−0.13 to 0.03), SIRI 0.04 (−0.45 to 0.67) (Table 6, Panel A). These intervals include differences in the size observed between participants who did and did not receive dialysis; therefore, the stratified comparisons do not establish the equivalence of the two groups.

Among participants with an eGFR of at least 60 mL/min/1.73 m^2^, the 14 with albuminuria-defined DKD (median eGFR 82.7; IQR 71.2 to 100.7) and the 62 with diabetes only did not differ in any index (NLR 1.95 versus 1.84, Hodges–Lehmann difference 0.05, 95% CI −0.42 to 0.53; MLR 0.26 versus 0.22, 0.02, −0.03 to 0.08; SIRI 1.02 versus 1.02 × 10^9^/L, −0.02, −0.31 to 0.34; *p* ≥ 0.31). This is the only contrast in the study that is free of renal function confounding; it is descriptive and has little power, but it provides no indication of an albuminuria-associated elevation of the indices.

## 4. Discussion

This study has two main findings. First, the indices were not specific to diabetes: all three indices reached their highest values in non-diabetic CKD, and within strata of comparable severity, including below an eGFR of 30 and 15 mL/min/1.73 m^2^ and among participants not receiving dialysis, the two nephropathy groups did not differ significantly. These strata were small (11 to 42 participants per group) and the confidence intervals for the differences were wide, so the absence of a significant difference is not evidence that the groups are equivalent; the strata do, however, show that the higher values in non-diabetic CKD were not reproduced once severity and dialysis were held comparable. All three indices were higher with dialysis, whereas the platelet count was not (209 vs. 221 × 10^9^/L; *p* = 0.21). Because 32 of 43 participants with non-diabetic CKD received dialysis, but only 12 of 66 with DKD, and because the non-diabetic group was almost entirely stage 5, the higher indices in non-diabetic CKD are more plausibly attributed to the severity of kidney disease and to dialysis-associated and uraemic inflammation than to cause. This imbalance is a feature of the sampling rather than of the biology, and it restricts the design to the question of specificity for diabetes. Model 3 makes the same point in an adjusted form: the cause was discriminated by eGFR alone, no index added information to a model containing it, including when eGFR was treated as not applicable in participants receiving dialysis, and NLR and MLR reached significance only when renal function was left uncontrolled, in the direction of a cause unrelated to diabetes. The odds ratio for eGFR in Model 3 and its high AUC largely reproduce the way the two groups were sampled and should not be read as a biological estimate.

The mechanisms underlying this pattern are reasonably well characterised. At each session, haemodialysis exposes blood to the dialyser membrane, which activates complement through the lectin and alternative pathways; the resulting anaphylatoxins stimulate leukocytes to release interleukin-6, tumour necrosis factor α and granule enzymes such as myeloperoxidase and elastase [23]. Between sessions, the retention of uraemic solutes and advanced glycation end products, exposure to dialysate endotoxin and the vascular access maintain a microinflammatory state [6,24]. The cellular consequence is asymmetric: myeloid cell numbers are preserved or increased, whereas T cells enter a state of persistent early activation with increased Fas (CD95) expression and are lost through activation-induced apoptosis, a process that is present in uraemia and intensified by haemodialysis itself [18,25]. Because the lymphocyte count is the denominator of all three indices, this asymmetry raises NLR, MLR and SIRI together, whereas the platelet count does not share this denominator and did not differ by dialysis status. In the present data, the lymphocyte count was lower with dialysis (1.31 versus 2.04 × 10^9^/L), whereas the monocyte count was unchanged, which is the pattern expected from uraemic lymphopenia rather than from monocyte activation. In dialysis populations, NLR correlates with interleukin-6 and tumour necrosis factor α (although the platelet-to-lymphocyte ratio tracks both more closely) and independently predicts mortality [26,27]. The correlation of NLR with these cytokines has a physiological basis. Proinflammatory cytokines such as interleukin-6 and tumour necrosis factor α, together with the cortisol and catecholamines released during systemic inflammation, mobilise neutrophils from the bone marrow and the marginated pool and delay their apoptosis, while they accelerate lymphocyte apoptosis and redistribute lymphocytes from the circulation to lymphoid tissue; NLR therefore integrates the opposing effects of a single cytokine milieu on the two lineages [28]. In end-stage kidney disease, this milieu is sustained because cytokine production is increased and renal clearance of cytokines is reduced [29]. Uraemic lymphopenia, acting on the shared denominator, is the signal underlying the use of NLR as an inflammation index in CKD and its association with endothelial dysfunction and cardiovascular events; a graded pattern has also been reported for the systemic immune inflammation index [30,31,32]. The association of NLR with endothelial dysfunction is best understood as the product of shared causes rather than of a direct effect of the ratio. Retained uraemic toxins such as indoxyl sulfate, together with chronic low-grade inflammation and oxidative stress, impair protective endothelial functions, including nitric oxide-dependent vasodilatation, and induce a proinflammatory and prothrombotic endothelial phenotype [33,34]; indoxyl sulfate also stimulates monocytes to produce tumour necrosis factor α, which recruits cytotoxic CD4+CD28− T cells that induce endothelial cell apoptosis [35]; and complement activation during haemodialysis primes neutrophils to release myeloperoxidase and elastase [23]. Each of these processes raises the neutrophil or monocyte count, lowers the lymphocyte count, or both, so that a high NLR marks the intensity of the process that injures the endothelium rather than causing the injury.

A similar pattern was observed in histological studies. Among 247 patients with biopsy-proven DKD, the NLR correlated inversely with eGFR and positively with interstitial fibrosis and tubular atrophy, but not with glomerular lesions or interstitial inflammation, pointing toward tubulointerstitial scarring rather than glomerular pathology specific to diabetes [36]. Two processes acting in opposite directions can account for these correlations. Tubulointerstitial fibrosis is the common structural correlate of the loss of glomerular filtration in progressive kidney disease of any cause; it is primed by non-resolving inflammation, in which infiltrating leukocytes and their cytokines activate matrix-producing fibroblasts, induce tubular atrophy, and cause microvascular rarefaction and hypoxia [37,38]. In DKD, this inflammation is driven by innate immune activation through Toll-like receptors, the NLRP3 inflammasome, and complement, which produces sterile tubulointerstitial inflammation and fibrosis [39]. A raised peripheral NLR may therefore reflect the systemic counterpart of intrarenal inflammation, which would explain its association with fibrosis and atrophy rather than with glomerular lesions. Conversely, as glomerular filtration falls, retained uraemic solutes depress the lymphocyte count through the mechanisms described above, so that part of the inverse correlation with eGFR is a consequence of renal impairment rather than a cause. Cross-sectional data cannot separate these two directions, and the inverse correlation of each index with eGFR among the participants with diabetes in the present study (ρ −0.31 to −0.37), carried mainly by the lymphocyte count, is compatible with both. Outside of diabetes, NLR was an independent determinant of tubular atrophy and interstitial fibrosis in 263 patients with IgA nephropathy (AUC 0.596) and predicted end-stage renal disease in 966 patients (AUC 0.633) [40,41]. Thus, modest discrimination is shared wherever the indices have been examined; the present four-group comparison is its clinical counterpart.

The second finding of our study concerns the incremental value, which depended on the comparator: no index added information to Model 1, and all three added information to Model 2. This difference was not incidental. Four of the five Model 1 covariates were correlated with eGFR, and creatinine alone discriminated nephropathy with an AUC of 0.931; a model reaching 0.803 without creatinine recovered much of the information in the case definition through its downstream consequences. Adjusting for anaemia, hyperuricaemia, and thrombocytopenia removes the variance that a marker of renal inflammation would be expected to explain; thus, the null is as much a property of the comparator as of the marker. The Model 2 covariates showed no such correlation, apart from age, and removing age strengthened this result. The LASSO-selected model, which retained nine covariates, yielded the same null as Model 1.

The results of Model 2 should nevertheless be interpreted with caution. Model 2 is a weak model (AUC 0.683) whose outcome is largely defined by eGFR, and the indices were correlated with eGFR among patients with diabetes (ρ −0.31 to −0.37), mainly through the lymphocyte count (ρ 0.30), whereas the neutrophil and monocyte counts were not. Any variable that tracks renal function would therefore be expected to improve Model 2, and the improvement is evidence that the indices carry renal signal rather than evidence of nephropathy-specific inflammation. The improvement was also fragile: only MLR survived Bonferroni correction, DeLong’s test did not detect the change in AUC, the optimism-corrected gain was at most 0.033, the calibration slopes of 0.77 to 0.78 indicated overfitting, and the signal was no longer significant once the 12 participants receiving dialysis were excluded, although the point estimates moved only modestly (for example, MLR 1.50 to 1.23 per 0.1) and the dialysis-excluded intervals overlap those of the full analysis. The covariates of Model 2 are, moreover, not wholly independent of renal function: HbA1c is lowered by anaemia, shortened red-cell survival and erythropoiesis-stimulating agents in advanced CKD, fasting glucose is affected by reduced insulin clearance, and age was correlated with eGFR (ρ −0.33). Finally, because creatinine and eGFR are always available in practice, Model 2 describes a research contrast rather than a clinical scenario: it shows that the indices contain information about renal impairment that is not captured by age, sex, and glycaemia, not that they would change the assessment of a patient whose eGFR is known.

The comparison with the component counts was inconclusive: within Model 2, the counts as a block did not improve the model, and each index added to its own components approached but did not reach significance (*p* = 0.065 to 0.071) in models with variance inflation factors above 5. These results do not show that the ratios summarise the counts more efficiently than the counts themselves; at most, they leave the question open. The only study to address this question directly reported that a high NLR predicted dialysis or death in CKD, whereas the counts separately did not, although with a dichotomised NLR and a longitudinal endpoint, so the correspondence is suggestive rather than exact [42].

The two findings jointly indicate that the indices reflect nephropathy but largely duplicate the information provided by the routine markers of renal impairment in the same laboratory panel. Clinically, Model 1 indicates that once haemoglobin, uric acid, and platelet count have been reviewed, calculating an index from the same blood count does not alter the assessment; for research, Model 2 indicates that the indices carry a signal that a study adjusting for the consequences of the outcome will fail to detect. This distinction has rarely been drawn explicitly, as the indices have generally been evaluated in isolation. The univariable results here reproduce those of others, including the systematic review and the largest available cohort cited above [10,12], the MADKID study, the association of MLR with interleukin-6 and carotid intima–media thickness, and studies of NLR alongside other inflammatory mediators in DKD [9,43,44].

Here, univariate discrimination (AUC 0.669 for MLR, 0.637 for SIRI, and 0.634 for NLR) was lower than that in studies that employed more extreme contrasts or stratified by albuminuria (0.874 for MLR; 0.869 for NLR): the MLR figure was derived from a comparison with patients free of any diabetic complication and the NLR figure from albuminuria strata, whereas the KDIGO definition used here combined participants with reduced eGFR and albuminuria and participants with albuminuria alone in a single group [8,13]. Apparent discrimination is also optimistically biased in single-centre studies; corrected values here fell by approximately 0.03 to 0.04, and a recent narrative review reached a similar conclusion regarding standalone performance [45]. The population-based NHANES analyses, in which SIRI and the systemic immune-inflammation index were associated with prevalent CKD and albuminuria and NLR with mortality in CKD, are consistent with the present interpretation that the indices track kidney disease and its severity rather than its cause [14,15,16].

The findings of the present study argue against the use of these indices as diabetes-specific markers of nephropathy: an elevated NLR, MLR, or SIRI in a person with diabetes is at least as consistent with kidney disease of any cause and with dialysis and should prompt the assessment of renal function rather than an inference about the cause. The same findings also argue that the incremental value of a composite index should be reported against a comparator free of the consequences of the outcome and separately against its component counts. The present findings do not address the clinically more pressing question of whether a person with diabetes and CKD has diabetic or non-diabetic kidney disease, which would require a cohort in which the cause of CKD is established independently of diabetes status, ideally through biopsy. Whether the indices predict progression remains unresolved; in a short follow-up study, NLR predicted a subsequent decline in kidney function among individuals with diabetes and prediabetes [46].

### Limitations

First, the single-centre, tertiary-care design may limit generalisability; although the protocol specified an eGFR of at least 90 mL/min/1.73 m^2^ for controls, 20 of the 60 had lower values (minimum 61.4), a protocol deviation described in Section 2.1, so the control group had preserved rather than supranormal renal function, and the sensitivity analysis excluding these participants is reported in Section 3.3; the controls were also predominantly obese (median BMI 30.2 kg/m^2^; 34 of 60 with a BMI of 30 or more), and obesity is itself associated with higher leukocyte counts and NLR, which would narrow rather than widen the contrast between controls and the other groups. Hypertension and cardiovascular disease were not exclusion criteria and were not recorded.

Second, the non-diabetic CKD group was predominantly dialysis-dependent and almost entirely stage 5, functioning as an advanced-CKD comparator rather than a stage-matched one; only 11 such participants were not receiving dialysis, so the comparisons that removed the effect of dialysis rested on 11 to 42 participants per group, and their confidence intervals were compatible with differences in clinically relevant size (Table 6). The imbalance in severity also means that Model 3 largely reproduces the sampling design: the adjustment for eGFR relies on extrapolation across groups with little overlap, and the odds ratio for eGFR and the AUC of 0.874 should not be interpreted as a biological estimate.

Third, creatinine-based eGFR is not valid in participants receiving maintenance dialysis, which depends on the timing of sampling relative to the session and the dialysis dose. Therefore, eGFR was used as a covariate in Model 3 and as a stratification variable only with this caveat; the alternative form of Model 3, which treats eGFR as not applicable during dialysis, gave the same result, and the eGFR strata below 15 mL/min/1.73 m^2^ should be read as mixing participants receiving and not receiving dialysis.

Fourth, the case definitions were clinical and no kidney biopsies were performed. Although persistent albuminuria was confirmed from the earlier records of all 52 participants with DKD who had a sustained eGFR below 60 mL/min/1.73 m^2^, in a population with a median age of 62 years and a presumably high prevalence of hypertension, some of these participants may have had hypertensive or ischaemic kidney disease coexisting with, or instead of, DKD, which would blur the contrast between the two nephropathy groups. Persistence of albuminuria rests on the records as described in the Methods; because the frequency and duration of albuminuria follow-up differed between patients, the assessment could not be standardised, and the albuminuria category could not be analysed. The non-diabetic CKD group was defined by the absence of diabetes, so Model 3 asks whether the indices distinguish CKD with diabetes from CKD without diabetes, not whether a person with diabetes has diabetic or non-diabetic kidney disease.

Fifth, the cross-sectional design precluded the assessment of progression. Sixth, 564 of the 957 screened records (59%) were excluded for incomplete laboratory data, the largest single reason for exclusion. Complete panels that include a leucocyte differential count are more likely to be available for patients under nephrology or dialysis follow-up, who undergo routine monthly blood counts, than for outpatients attending for a routine check, so the analysed sample may over-represent more closely monitored patients, and the group comparisons may be affected by selection bias of unknown magnitude because a comparison of the excluded with the included records was not undertaken. Continuous albuminuria could not be analysed for the reason given above, and diabetes duration, C-reactive protein, smoking, and medication data were not retrievable; diabetes duration is a particularly plausible confounder, and its absence is the main reason Model 2 discriminates only modestly, since HbA1c did not differ between the diabetes groups. The direction of the resulting biases can be anticipated. Sodium–glucose cotransporter 2 inhibitors, glucagon-like peptide 1 receptor agonists, statins, and renin–angiotensin system blockers lower inflammatory markers and are prescribed more often to people with DKD than to people with diabetes alone, which would bias the DKD–diabetes-only contrast toward the null; metformin, which also lowers inflammatory markers, is withdrawn at low eGFR and would therefore act in the opposite direction; erythropoiesis-stimulating agents and intravenous iron, confined to the dialysis-dependent participants, affect haemoglobin (a Model 1 covariate) and possibly leukocyte subsets, and would contribute to the dialysis effect; and smoking raises the neutrophil count, with an effect on the group contrasts that depends on its unknown distribution across groups.

Seventh, Model 1′s selection step is data-driven, and the bootstrap held its composition fixed; therefore, its corrected figures remain optimistic, although the LASSO-selected model gave the same null; Model 2, defined on causal grounds, was unaffected. No protocol or statistical analysis plan was registered, and although the analytical roles of the variables were fixed before the indices were analysed, the number of models, sensitivity analyses, and subgroup comparisons means that chance findings cannot be excluded. Eighth, the Model 2 result is fragile for the reasons set out in the Discussion (multiplicity, the DeLong test, optimism correction, and the dialysis-excluded subset) and should be regarded as a signal requiring replication. The Model 3 null should likewise be read as the absence of a moderate effect rather than equivalence; the confidence intervals of the adjusted odds ratios (Table 4, Panel F) show the range of effects that are compatible with the data, and effects outside these intervals are those the data argue against. Finally, the analyses were internally but not externally validated, and corrected calibration slopes of 0.75 to 0.86 indicated residual overfitting.

## 5. Conclusions

In this single-centre cohort, NLR, MLR, and SIRI were highest in a non-diabetic CKD group that was almost entirely stage 5 and predominantly dialysis-dependent; were higher in participants receiving dialysis; and did not differ significantly between diabetic and non-diabetic kidney disease within small strata of comparable severity, a pattern more consistent with the severity of kidney disease and its treatment than with a process specific to diabetes. Their incremental value depended on the comparator: no index added information to a model containing routinely available markers of renal impairment, whereas all three added information to a model restricted to age, sex, and glycaemia, with the qualifications that only MLR survived Bonferroni correction, the optimism-corrected gain in discrimination was small, and the signal was no longer significant after participants receiving dialysis were excluded. The indices therefore appear redundant with, rather than uninformative beyond, the routine markers of renal impairment reported alongside them. The design cannot determine whether a person with diabetes has diabetic or non-diabetic kidney disease. Prospective, multicentre, stage-matched studies with longitudinal follow-up are needed to confirm these observations and to determine whether the indices carry prognostic information.

## Figures and Tables

**Table 1:** Baseline characteristics of the participants.

Characteristic	Controls (*N* = 60)	Diabetes Only (*N* = 63)	Diabetic Kidney Disease (*N* = 66)	Non-Diabetic CKD (*N* = 43)	*p* Value	ε^2^
Female sex—no. (%)	39 (65.0)	42 (66.7)	33 (50.0)	22 (51.2)	0.13	—
Age—years	53.0 (48.8–60.0)	56.0 (49.5–65.5)	62.0 (56.0–71.0)	56.0 (46.0–66.0)	0.002	0.051
Receiving dialysis—no. (%)	0	0	12 (18.2)	32 (74.4)	<0.001	—
KDIGO G category—no. (%) §						
G1 (eGFR ≥ 90)	40 (66.7)	44 (69.8)	6 (9.1)	0	—	—
G2 (60–89)	20 (33.3)	19 (30.2)	8 (12.1)	1 (2.3)		
G3a (45–59)	0	0	11 (16.7)	0		
G3b (30–44)	0	0	11 (16.7)	0		
G4 (15–29)	0	0	10 (15.2)	2 (4.7)		
G5 (<15), not receiving dialysis	0	0	8 (12.1)	8 (18.6)		
G5D (receiving dialysis)	0	0	12 (18.2)	32 (74.4)		
Body mass index—kg/m^2^ †	30.2 (26.4–34.4)	30.8 (27.8–36.3)	30.2 (27.4–31.7)	25.0 (22.6–30.5)	<0.001	0.065
Waist circumference—cm	102.0 (96.8–110.0)	108.0 (98.0–114.5)	104.7 (99.2–109.8)	101.0 (93.5–109.0)	0.078	0.017
Complete blood count						
White-cell count—×10^9^/L	6.60 (5.82–7.81)	8.26 (6.83–9.09)	8.00 (6.53–9.21)	6.32 (5.52–7.77)	<0.001	0.111
Neutrophils—×10^9^/L	3.66 (3.20–4.10)	4.69 (3.99–5.57)	4.76 (3.73–6.14)	4.16 (3.52–4.85)	<0.001	0.115
Lymphocytes—×10^9^/L	2.21 (1.82–3.00)	2.56 (1.83–2.94)	2.04 (1.37–2.67)	1.32 (1.12–1.69)	<0.001	0.203
Monocytes—×10^9^/L	0.51 (0.41–0.61)	0.56 (0.47–0.66)	0.59 (0.49–0.72)	0.50 (0.45–0.63)	0.017	0.032
Haemoglobin—g/dL	13.8 (13.1–15.2)	14.1 (13.1–15.1)	12.9 (11.4–14.5)	11.5 (10.8–12.6)	<0.001	0.237
Haematocrit—%	40.8 (39.1–44.4)	42.0 (39.0–43.9)	39.1 (34.4–43.0)	34.6 (32.1–37.2)	<0.001	0.211
Platelet count—×10^9^/L	239 (212–298)	267 (210–324)	226 (193–267)	208 (168–248)	<0.001	0.075
Mean platelet volume—fL	10.2 (9.7–10.9)	10.3 (9.8–10.9)	10.4 (9.7–11.1)	10.4 (9.8–11.1)	0.739	0.000
Biochemical measurements						
Fasting glucose—mg/dL	95 (89–102)	144 (118–199)	146 (122–224)	102 (92–128)	<0.001	0.387
HbA1c—% ‡	5.6 (5.4–6.0)	7.7 (6.5–9.4)	7.3 (6.8–9.2)	5.4 (5.1–5.6)	<0.001	0.651
Uric acid—mg/dL	5.3 (4.3–5.6)	5.1 (4.3–5.7)	6.0 (5.1–6.8)	5.6 (5.0–6.0)	<0.001	0.060
Aspartate aminotransferase—U/L	17.5 (15.0–21.0)	16.0 (13.8–20.1)	17.0 (13.0–21.5)	11.8 (9.1–17.6)	<0.001	0.070
Alanine aminotransferase—U/L	17.0 (12.5–24.0)	17.0 (13.3–25.0)	15.1 (11.6–19.8)	9.0 (7.5–17.5)	<0.001	0.086
Creatinine—mg/dL	0.72 (0.63–0.90)	0.75 (0.60–0.90)	1.75 (1.22–4.05)	7.60 (5.54–9.70)	<0.001	—¶
eGFR—mL/min/1.73 m^2^, all participants	102.2 (86.8–107.5)	101.2 (85.9–109.0)	35.5 (12.5–57.3)	7.4 (5.2–9.5)	<0.001	—¶
eGFR—mL/min/1.73 m^2^, participants not receiving dialysis (*N*)	102.2 (86.8–107.5) (60)	101.2 (85.9–109.0) (63)	40.4 (21.9–59.7) (54)	11.5 (10.2–16.6) (11)	<0.001	—¶

Data are medians (interquartile ranges) unless otherwise indicated. *p* values were calculated using the Kruskal–Wallis test for continuous variables and the chi-square test for categorical variables. ε^2^ denotes the epsilon-squared effect size (0.01 indicates a small effect; 0.06, a moderate effect; and 0.14, a large effect) and is not defined for categorical variables. To convert glucose to mmol/L, multiply by 0.0555; creatinine to μmol/L, by 88.4; and uric acid to μmol/L, by 59.48. CKD, chronic kidney disease; eGFR, estimated glomerular filtration rate; HbA1c, glycated haemoglobin; KDIGO, Kidney Disease: Improving Global Outcomes. † Body mass index was calculated as weight in kilograms divided by the square of the height in metres. ‡ HbA1c is reported in NGSP percentage units; to convert to IFCC mmol/mol, multiply by 10.93 and subtract 23.5. § G categories follow the KDIGO 2024 [21] classification; participants receiving maintenance dialyswere classified as G5D irrespective of their eGFR, because creatinine-based eGFR is not valid during dialysis. Diabetes duration, smoking status, and medication use were not retrievable from the records; the albuminuria category is could not be assessed because the frequency and duration of albuminuria follow-up differed between patients and could not be standardised. ¶ Not reported: creatinine and eGFR define group membership; therefore, an effect size for the four-group comparison is not informative.

**Table 2:** Composite inflammatory indices according to study group.

Index	Controls (*N* = 60)	Diabetes Only (*N* = 63)	Diabetic Kidney Disease (*N* = 66)	Non-Diabetic CKD (*N* = 43)	*p* Value	ε^2^	Adjusted Test †	Adjusted *p* Value	Partial η^2^
NLR	1.59 (1.20–2.11)	1.84 (1.46–2.39)	2.33 (1.63–3.91)	3.43 (2.27–4.26)	<0.001	0.182	F(3, 226) = 16.36	<0.001	0.178
MLR	0.21 (0.17–0.29)	0.22 (0.17–0.31)	0.29 (0.23–0.41)	0.38 (0.29–0.54)	<0.001	0.182	F(3, 226) = 16.66	<0.001	0.181
SIRI—×10^9^/L	0.76 (0.61–1.08)	1.02 (0.74–1.44)	1.28 (0.97–2.35)	1.70 (1.09–2.48)	<0.001	0.181	F(3, 226) = 15.36	<0.001	0.169

Data are medians (interquartile ranges). *p* values and ε^2^ are from the Kruskal–Wallis test. Dunn’s post hoc comparisons with Bonferroni correction are shown in Figure 2. CKD, chronic kidney disease; MLR, monocyte-to-lymphocyte ratio; NLR, neutrophil-to-lymphocyte ratio; SIRI, systemic inflammation response index. † The adjusted test was a rank analysis of covariance, with age and sex as covariates.

**Table 3:** Univariate associations with diabetic kidney disease among participants with diabetes.

Variable	Odds Ratio (95% CI)	*p* Value
Age—per 10 years	1.57 (1.10–2.24)	0.013
Female sex	0.50 (0.25–1.02)	0.056
Body mass index—per kg/m^2^	0.93 (0.87–1.00)	0.041
Waist circumference—per cm	0.97 (0.94–1.01)	0.105
Fasting glucose—per 10 mg/dL	1.02 (0.98–1.06)	0.341
HbA1c—per 1% (11 mmol/mol)	0.99 (0.84–1.17)	0.936
Uric acid—per mg/dL	1.53 (1.16–2.00)	0.002
Aspartate aminotransferase—per U/L	1.02 (0.98–1.07)	0.335
Alanine aminotransferase—per U/L	0.99 (0.96–1.02)	0.611
Haemoglobin—per g/dL	0.75 (0.63–0.91)	0.003
Haematocrit—per %	0.91 (0.85–0.97)	0.005
Platelet count—per 10 × 10^9^/L	0.92 (0.87–0.97)	0.003
Mean platelet volume—per fL	1.20 (0.80–1.81)	0.383
Components of the indices †		
White-cell count—per ×10^9^/L	1.00 (0.84–1.19)	0.994
Neutrophils—per ×10^9^/L	1.12 (0.89–1.40)	0.321
Lymphocytes—per ×10^9^/L	0.59 (0.39–0.90)	0.014
Monocytes—per 0.1 × 10^9^/L	1.14 (0.93–1.40)	0.205
Constituents of the case definition †		
Creatinine—per 0.1 mg/dL	1.87 (1.47–2.38)	<0.001
eGFR—per mL/min/1.73 m^2^	0.92 (0.90–0.95)	<0.001
Composite inflammatory indices		
NLR—per 1	1.62 (1.18–2.22)	0.003
MLR—per 0.1	1.67 (1.22–2.29)	0.002
SIRI—per 1 × 10^9^/L	1.96 (1.23–3.11)	0.004

Diabetic kidney disease (*N* = 66) versus diabetes without nephropathy (*N* = 63). Odds ratios are per the increment shown. Age is expressed per 10 years and creatinine per 0.1 mg/dL so that the odds ratios are on a clinically interpretable scale; the wide interval for creatinine reflects the near-complete separation of the groups by a variable that enters the case definition. CI, confidence interval; eGFR, estimated glomerular filtration rate; HbA1c, glycated haemoglobin; MLR, monocyte-to-lymphocyte ratio; NLR, neutrophil-to-lymphocyte ratio; SIRI, systemic inflammation response index. † These variables are shown for completeness but were excluded from both the comparator models by design.

**Table 4:** Incremental value of each index over the three comparator models.

*Panel A. Model 1, the conventional comparator (χ^2^ = 40.46, df = 5, p < 0.001; Nagelkerke R^2^ = 0.359; AUC 0.803, 95% CI 0.728–0.872; 12.6 events per variable)*										
Variable						Adjusted odds ratio (95% CI)			*p* value	
Age—per 10 years						1.25 (0.83–1.87)			0.288	
Body mass index—per kg/m^2^						0.91 (0.84–0.99)			0.023	
Uric acid—per mg/dL						1.65 (1.16–2.35)			0.005	
Haemoglobin—per g/dL						0.70 (0.56–0.87)			0.001	
Platelet count—per 10 × 10^9^/L						0.92 (0.87–0.98)			0.007	
*Panel B. Each index added to Model 1 as a separate second block*										
Index added	Adjusted OR (95% CI)	Δχ^2^	*p* value		Bonferroni *p*	BH q	ΔR^2^	AUC (95% CI)	ΔAUC	DeLong *p*
SIRI—per 1 × 10^9^/L	1.50 (0.93–2.42)	3.16	0.076		0.227	0.188	+0.024	0.810 (0.733–0.877)	+0.007	0.56
NLR—per 1	1.31 (0.92–1.86)	2.35	0.125		0.376	0.188	+0.018	0.806 (0.729–0.877)	+0.003	0.77
MLR—per 0.1	1.20 (0.84–1.71)	1.05	0.305		0.915	0.305	+0.008	0.803 (0.725–0.875)	+0.000	0.98
*Panel C. Model 2, restricted to variables not determined by renal function † (χ^2^ = 12.84, df = 4, p = 0.012; Nagelkerke R^2^ = 0.126; AUC 0.683, 95% CI 0.583–0.768; 15.8 events per variable)*										
Variable						Adjusted odds ratio (95% CI)			*p* value	
Age—per 10 years						1.61 (1.12–2.31)			0.011	
Female sex						0.48 (0.23–1.01)			0.053	
HbA1c—per 1% (11 mmol/mol)						0.88 (0.68–1.13)			0.318	
Fasting glucose—per 10 mg/dL						1.05 (0.98–1.12)			0.140	
*Panel D. Each index added to Model 2 as a separate second block*										
Index added	Adjusted OR (95% CI)	Δχ^2^	*p* value		Bonferroni *p*	BH q	ΔR^2^	AUC (95% CI)	ΔAUC	DeLong *p*
MLR—per 0.1	1.50 (1.07–2.10)	6.61	0.010		0.030	0.029	+0.060	0.718 (0.622–0.803)	+0.035	0.22
SIRI—per 1 × 10^9^/L	1.60 (1.01–2.54)	4.87	0.027		0.082	0.029	+0.045	0.700 (0.604–0.783)	+0.018	0.44
NLR—per 1	1.43 (1.02–2.00)	4.74	0.029		0.088	0.029	+0.044	0.703 (0.605–0.788)	+0.021	0.41
*Panel E. Model 3 ‡, confined to the participants with nephropathy of either cause (N = 109), with the cause of the nephropathy as the outcome (χ^2^ = 51.41, df = 4, p < 0.001; Nagelkerke R^2^ = 0.509; AUC 0.874, 95% CI 0.795–0.936; 10.8 events per variable)*										
Variable						Adjusted odds ratio (95% CI)			*p* value	
Age—per 10 years						1.17 (0.78–1.75)			0.437	
Female sex						0.91 (0.33–2.51)			0.862	
eGFR—per 10 mL/min/1.73 m^2^						2.25 (1.20–4.20)			0.011	
Receiving dialysis						0.43 (0.12–1.59)			0.208	
*Panel F. Each index added to Model 3 as a separate second block*										
Index added	Adjusted OR (95% CI)	Δχ^2^	*p* value		Bonferroni *p*	BH q	ΔR^2^	AUC (95% CI)	ΔAUC	DeLong *p*
SIRI—per 1 × 10^9^/L	1.14 (0.73–1.77)	0.32	0.569		1.000	0.913	+0.003	0.870 (0.792–0.933)	−0.005	0.48
NLR—per 1	0.98 (0.71–1.35)	0.02	0.888		1.000	0.913	+0.000	0.873 (0.795–0.936)	−0.001	0.75
MLR—per 0.1	1.02 (0.74–1.40)	0.01	0.913		1.000	0.913	+0.000	0.876 (0.797–0.937)	+0.001	0.41
*Panel G. Model 3 with eGFR treated as not applicable in participants receiving dialysis (age, sex, dialysis indicator, and eGFR entered only for participants not receiving dialysis; χ^2^ = 49.94, df = 4; Nagelkerke R^2^ = 0.498; AUC 0.862)*										
Variable or index added				Adjusted OR (95% CI)		Δχ^2^	*p* value	AUC with index	ΔAUC	
Age—per 10 years				1.18 (0.79–1.77)		—	0.415	—	—	
Female sex				0.86 (0.31–2.33)		—	0.760	—	—	
Receiving dialysis (eGFR not applicable)				0.63 (0.13–3.00)		—	0.566	—	—	
eGFR among participants not receiving dialysis—per 10 mL/min/1.73 m^2^				2.07 (1.16–3.71)		—	0.014	—	—	
SIRI—per 1 × 10^9^/L				1.11 (0.71–1.73)		0.22	0.640	0.857	−0.004	
NLR—per 1				0.96 (0.70–1.32)		0.06	0.810	0.862	0.000	
MLR—per 0.1				1.00 (0.73–1.37)		0.00	0.984	0.862	0.000	

Events per variable are based on the smaller of the two outcome groups. Δχ^2^ is the likelihood ratio test for the added index (1 df), and the *p* value is its nominal significance; the Bonferroni *p* value and BH q value are corrected for the three indices tested within the same model. ΔR^2^ is the change in Nagelkerke’s R^2^, and ΔAUC the change in the area under the curve, with paired AUCs compared by DeLong’s test. Odds ratios are adjusted for every covariate in the model. AUC, area under the receiver operating characteristic curve; BH, Benjamini–Hochberg; CI, confidence interval; eGFR, estimated glomerular filtration rate; HbA1c, glycated haemoglobin; MLR, monocyte-to-lymphocyte ratio; NLR, neutrophil-to-lymphocyte ratio; OR, odds ratio; SIRI, systemic inflammation response index. † Among the participants with diabetes, the Spearman correlation with eGFR was −0.42 for uric acid, +0.40 for haemoglobin, and +0.23 for platelet count (*p* ≤ 0.007 for all), and +0.16 for body mass index (*p* = 0.068), against +0.10 for sex (*p* = 0.27), +0.13 for HbA1c (*p* = 0.15) and −0.02 for fasting glucose (*p* = 0.79); age (ρ = −0.33) was common to both models. ‡ Models 1 and 2 were fitted to 129 participants with diabetes, in whom the outcome was the presence of diabetic kidney disease (66 events). Model 3 was fitted in 109 participants who already had nephropathy, in whom the outcome was its cause (66 diabetic and 43 non-diabetic; the smaller group governed the events per variable). Because the case definition in Model 3 was the presence of diabetes rather than the level of renal function, eGFR and dialysis status were confounders rather than circular constituents and were adjusted for; HbA1c and fasting glucose were excluded from Model 3 as definitional. Panel G shows an alternative form of Model 3, in which creatinine-based eGFR was not applicable in participants receiving dialysis.

**Table 5:** Internal validation and analysis excluding participants receiving dialysis.

*Panel A. Optimism-corrected performance (Models 1 and 2, N = 129; Model 3, N = 109)*										
Model		Apparent AUC		Corrected AUC		Corrected ΔAUC		Calibration slope		
Model 1		0.803		0.776		—		0.856		
Model 1 + SIRI		0.810		0.779		+0.003		0.837		
Model 1 + NLR		0.806		0.774		−0.002		0.832		
Model 1 + MLR		0.803		0.771		−0.005		0.833		
Model 2		0.683		0.644		—		0.780		
Model 2 + MLR		0.718		0.676		+0.033		0.777		
Model 2 + NLR		0.703		0.660		+0.016		0.768		
Model 2 + SIRI		0.700		0.658		+0.014		0.766		
Model 3		0.874		0.855		—		0.781		
Model 3 + MLR		0.876		0.850		−0.005		0.754		
Model 3 + NLR		0.873		0.847		−0.008		0.753		
Model 3 + SIRI		0.870		0.845		−0.010		0.756		
*Panel B. Incremental value after exclusion of participants receiving dialysis (N = 117; 54 events; Model 1 AUC 0.789; Model 2 AUC 0.724)*										
Index added	Model 1 adjusted OR (95% CI)		Model 1 Δχ^2^		Model 1 *p* value		Model 2 adjusted OR (95% CI)		Model 2 Δχ^2^	Model 2 *p* value
NLR—per 1	1.27 (0.88–1.83)		1.62		0.204		1.32 (0.92–1.90)		2.36	0.124
MLR—per 0.1	1.06 (0.73–1.55)		0.09		0.765		1.23 (0.87–1.74)		1.47	0.226
SIRI—per 1 × 10^9^/L	1.41 (0.88–2.25)		2.12		0.145		1.44 (0.92–2.24)		2.86	0.091

The apparent values were obtained in the sample used to fit the model; corrected values followed Harrell’s bootstrap optimism procedure with 1000 replicates. The bootstrap was run with the model composition held fixed; for Model 1, whose composition was derived from these data by univariable screening, the corrected values therefore remained optimistic; Model 2 was defined on causal grounds and was not affected. Model 1 comprises age, body mass index, uric acid, haemoglobin, and platelet count; Model 2 comprises age, sex, HbA1c, and fasting glucose; Model 3 comprises age, sex, eGFR, and dialysis status and was fitted in 109 participants with nephropathy of either cause. In Panel B, Δχ^2^ is the likelihood-ratio test for the added index (1 df), and odds ratios are adjusted for every covariate in the respective model. AUC, area under the receiver-operating-characteristic curve; CI, confidence interval; eGFR, estimated glomerular filtration rate; HbA1c, glycated haemoglobin; MLR, monocyte-to-lymphocyte ratio; NLR, neutrophil-to-lymphocyte ratio; OR, odds ratio; SIRI, systemic inflammation response index.

**Table 6:** Effect sizes for two-group contrasts and correlations of the indices with eGFR.

*Panel A. Hodges–Lehmann median differences with 95% confidence intervals*							
Contrast (first minus second group)		*N*	NLR		MLR		SIRI (×10^9^/L)
DKD vs. non-diabetic CKD, all participants		66 vs. 43	−0.63 (−1.25 to −0.06); *p* = 0.028		−0.09 (−0.14 to −0.03); *p* = 0.006		−0.21 (−0.57 to 0.15); *p* = 0.24
Dialysis vs. no dialysis, nephropathy of either cause		44 vs. 65	0.79 (0.19 to 1.46); *p* = 0.010; rrb 0.29		0.12 (0.06 to 0.19); *p* < 0.001; rrb 0.46		0.37 (0.02 to 0.74); *p* = 0.034; rrb 0.24
DKD vs. non-diabetic CKD, eGFR < 30 mL/min/1.73 m^2^		30 vs. 42	−0.29 (−1.07 to 0.46); *p* = 0.37		−0.04 (−0.11 to 0.04); *p* = 0.40		0.01 (−0.46 to 0.44); *p* = 0.94
DKD vs. non-diabetic CKD, eGFR < 15 mL/min/1.73 m^2^		20 vs. 40	−0.33 (−1.27 to 0.52); *p* = 0.34		−0.01 (−0.11 to 0.07); *p* = 0.77		0.02 (−0.54 to 0.45); *p* = 0.91
DKD vs. non-diabetic CKD, not receiving dialysis		54 vs. 11	−0.10 (−0.85 to 0.84); *p* = 0.73		−0.06 (−0.13 to 0.03); *p* = 0.15		0.04 (−0.45 to 0.67); *p* = 0.87
Albuminuria-defined DKD vs. diabetes only, eGFR ≥ 60 mL/min/1.73 m^2^		14 vs. 62	0.05 (−0.42 to 0.53); *p* = 0.80		0.02 (−0.03 to 0.08); *p* = 0.31		−0.02 (−0.31 to 0.34); *p* = 0.88
*Panel B. Spearman correlation with eGFR*							
Variable	All participants with diabetes (*N* = 129)			Participants with diabetes not receiving dialysis (*N* = 117)		Participants with DKD (*N* = 66)	
NLR	−0.31 (*p* < 0.001)			−0.30 (*p* = 0.001)		−0.24 (*p* = 0.054)	
MLR	−0.37 (*p* < 0.001)			−0.25 (*p* = 0.006)		−0.37 (*p* = 0.002)	
SIRI	−0.34 (*p* < 0.001)			−0.29 (*p* = 0.002)		−0.29 (*p* = 0.016)	
Lymphocyte count	+0.30 (*p* < 0.001)			+0.23 (*p* = 0.013)		+0.23 (*p* = 0.058)	
Neutrophil count	−0.06 (*p* = 0.51)			−0.19 (*p* = 0.043)		−0.02 (*p* = 0.88)	
Monocyte count	−0.14 (*p* = 0.10)			−0.09 (*p* = 0.32)		−0.13 (*p* = 0.30)	
White-cell count	+0.02 (*p* = 0.80)			−0.09 (*p* = 0.36)		+0.02 (*p* = 0.86)	

Panel A shows the Hodges–Lehmann estimate of the median difference (first group minus second group) with a distribution-free 95% confidence interval, the Mann–Whitney *p* value, and, for the dialysis contrast, the rank-biserial correlation (rrb). The eGFR strata use creatinine-based eGFR, which is not valid in participants receiving dialysis; the stratum below 15 mL/min/1.73 m^2^ therefore mixes participants receiving and not receiving dialysis, and the contrast among participants not receiving dialysis is the only one unaffected by this. CKD, chronic kidney disease; DKD, diabetic kidney disease; eGFR, estimated glomerular filtration rate; MLR, monocyte-to-lymphocyte ratio; NLR, neutrophil-to-lymphocyte ratio; SIRI, systemic inflammation response index.

## Data Availability

The de-identified participant-level dataset and analysis codes are available from the corresponding author upon reasonable requests.

## Abbreviations

The following abbreviations are used in this manuscript.

AUC	Area under the receiver-operating-characteristic curve
BH	Benjamini–Hochberg
BMI	Body mass index
CI	Confidence interval
CKD	Chronic kidney disease
CKD-EPI	Chronic Kidney Disease Epidemiology Collaboration
DKD	Diabetic kidney disease
DM	Diabetes mellitus
eGFR	Estimated glomerular filtration rate
EPV	Events per variable
HbA1c	Glycated haemoglobin
IQR	Interquartile range
KDIGO	Kidney Disease: Improving Global Outcomes
LASSO	Least absolute shrinkage and selection operator
MLR	Monocyte-to-lymphocyte ratio
NLR	Neutrophil-to-lymphocyte ratio
NLRP3	NOD-like receptor family pyrin domain-containing 3
OR	Odds ratio
rrb	Rank-biserial correlation
SIRI	Systemic inflammation response index
uACR	Urinary albumin-to-creatinine ratio
